# *Lactobacillus amylovorus* KU4 inhibits adipocyte senescence in aged mice through necdin regulation of p53 activity

**DOI:** 10.18632/aging.206314

**Published:** 2025-09-03

**Authors:** Garam Yang, Eunjeong Hong, Hyuno Kang, Sejong Oh, Eungseok Kim

**Affiliations:** 1 Department of Biological Sciences, College of Natural Sciences, Chonnam National University, Buk-gu, Gwangju 61186, Republic of Korea; 2 Metropolitan Seoul Center, Korea Basic Science Institute, Seongbuk-gu, Seoul 02841, Republic of Korea; 3 Division of Animal Science, College of Agriculture and Lifesciences, Chonnam National University, Gwangju 61186, Republic of Korea

**Keywords:** Lactobacillus amylovorus KU4, adipocyte senescence, necdin, p53, aging

## Abstract

Previously, we reported that *Lactobacillus amylovorus* KU4 (LKU4) ameliorates diet-induced metabolic disorders by regulating adipose tissue (AT) physiology. Since metabolic disorders and age-related pathological conditions mutually exacerbate each other, this study hypothesizes that LKU4 may protect against adipose senescence during aging. Thus, this study demonstrates that LKU4 administration suppresses age-related metabolic dysfunction and aging phenotypes in AT of 24-month-old mice. Furthermore, LKU4 suppressed the expression of senescence marker genes, including *p53*, in the AT of these mice in parallel with the upregulation of *necdin* (*NDN*). Particularly, the effect of LKU4 on the expression of these genes was enhanced in adipocytes compared to stromal vascular fraction (SVF) cells. Mechanistically, NDN mediates the LKU4-induced suppression of *p53* transcriptional activity by blocking the p53–p300 interaction, thereby inhibiting p53 acetylation. Both LKU4 and NDN consistently reduced the senescence-associated secretory phenotype (SASP) in the AT of aged mice and senescent 3T3-L1 adipocytes. Furthermore, *ex vivo* NDN silencing in the AT of D-galactose-induced aging mice abolished LKU4 protection against p53-induced adipose senescence, reducing adipogenesis and mitochondrial dysfunction in primary adipocytes. These findings demonstrate that LKU4 inhibits age-induced adipocyte senescence by modulating the p53–p300 interaction through NDN, thereby protecting against age-associated metabolic disorders.

## INTRODUCTION

Aging represents a gradual decline in tissue and organ homeostasis over time, primarily driven by constituent cell dysfunction. Meanwhile, chronic stimulation of diverse cellular stressors leads to various forms of cellular damage, such as genomic instability, mitochondrial dysfunction, and telomere attrition, contributing to cellular senescence—a state of permanent arrest in the cell cycle.

Progressive accumulation of senescent cells leads to tissue dysfunction and inflammation through the secretion of the senescence-associated secretory phenotype (SASP), a complex network of molecules that includes proinflammatory and matrix-degrading factors [1]. The SASP can reprogram neighboring and distant cells by altering the cellular microenvironment, ultimately disrupting the physiological homeostasis.

Adipose tissue (AT) plays a major role in energy storage and mobilization through dynamic tissue remodeling in response to energy demands. Adipocytes are the principal adipose cells for AT homeostasis and also function as endocrine cells by secreting adipokines, including various inflammatory cytokines. Therefore, adipocyte dysfunction leads to excessive lipid and cytokine secretion, driving the aging process and associated metabolic disorders [2, 3]. Cellular senescence in AT increases under metabolic alterations such as obesity, diabetes, and insulin resistance [4]. Aging impairs the metabolic function of AT by disrupting the diverse cell populations, including progenitor cells, mature adipocytes, microvascular endothelial cells, and immune cells [5]. Moreover, mature adipocytes, constituting 20–40% of AT resident cells, can enter a senescence state. Age-associated senescence in these cells increases the SASP secretion, which impairs AT remodeling by inhibiting preadipocyte differentiation and promoting the accumulation of senescent adipocytes. The subsequent accumulation of senescent adipocytes exacerbates chronic inflammation through persistent SASP secretion and abnormal lipid accumulation [6]. This excess lipid storage in the AT leads to ectopic lipid deposition in the liver and skeletal muscle, further promoting systemic insulin resistance and chronic inflammation [7].

Chronic cellular stressors, including oxidative and metabolic stresses, promote sustained p53 activation in the AT, which induces irreversible cell cycle arrest in adipose progenitor cells and stimulates cellular senescence, contributing to tissue dysfunction and aging [8]. In aged white adipose tissue (WAT), increased p53 expression induces the expression of p21 and other cell cycle inhibitors; moreover, increased p53 expression promotes the production of proinflammatory cytokines, causing insulin resistance [9].

Many studies have shown that certain probiotic strains promote healthy aging by maintaining metabolic homeostasis and reducing age-related chronic inflammation [10–12]. However, despite the well-established role of cellular senescence in aging, the pathways through which probiotic bacteria influence cellular senescence remain largely unexplored. Recently, we reported that the administration of *Lactobacillus amylovorus* KU4 (LKU4) improves metabolic parameters and insulin resistance in diet-induced obese mice [13, 14]. Notably, metabolic abnormalities such as obesity are well-known triggers of cellular senescence in AT and are also closely associated with the aging process. These findings suggest that LKU4 may regulate cellular senescence in AT under aging conditions.

*NDN* is a gene that encodes a MAGE family protein. Meanwhile, NDN has been known to directly interact with p53 and regulate p53 activity by facilitating SIRT1-induced deacetylation of p53 in response to DNA damage [15]. NDN levels are abundant in WAT but decline significantly with age. However, the role of NDN in adipose senescence remains unknown. Interestingly, the NDN binding site within the N-terminal transactivation domain 2 (TAD2) of p53 overlaps with the binding site of p300 acetyl transferase, a p53 coactivator [16]. These findings suggest that NDN may regulate adipocyte senescence by inhibiting p53 activity through blocking p300-induced acetylation.

This study found that LKU4 administration attenuates adipocyte senescence in aged WAT by increasing the expression of NDN while downregulating p53 activity. Moreover, LKU4 reduced p300-induced acetylation of p53 in adipocytes under senescence conditions through NDN-mediated inhibition of the p53–p300 interaction, which occurs in a SIRT1-independent manner, consequently mitigating adipocyte senescence and SASP secretion. Our results demonstrate that LKU4 is critical for maintaining homeostasis in AT during aging, suggesting a potential application for LKU4 administration in age-related metabolic dysfunction.

## RESULTS

### LKU4 reduces cellular senescence in WAT of aged mice

Subcutaneous AT serves as a primary site for safely storing excess energy. However, inguinal WAT (iWAT) is prone to age-related alterations such as senescence and genomic instability [17]. To determine the effect of LKU4 on age-related adipose senescence in iWAT, 10-month-old C57BL/6J male mice were orally supplemented with LKU4 or vehicle (PBS) for 14 months, designated as aged LKU4 mice and control-aged mice, respectively.

**Figure 1:**
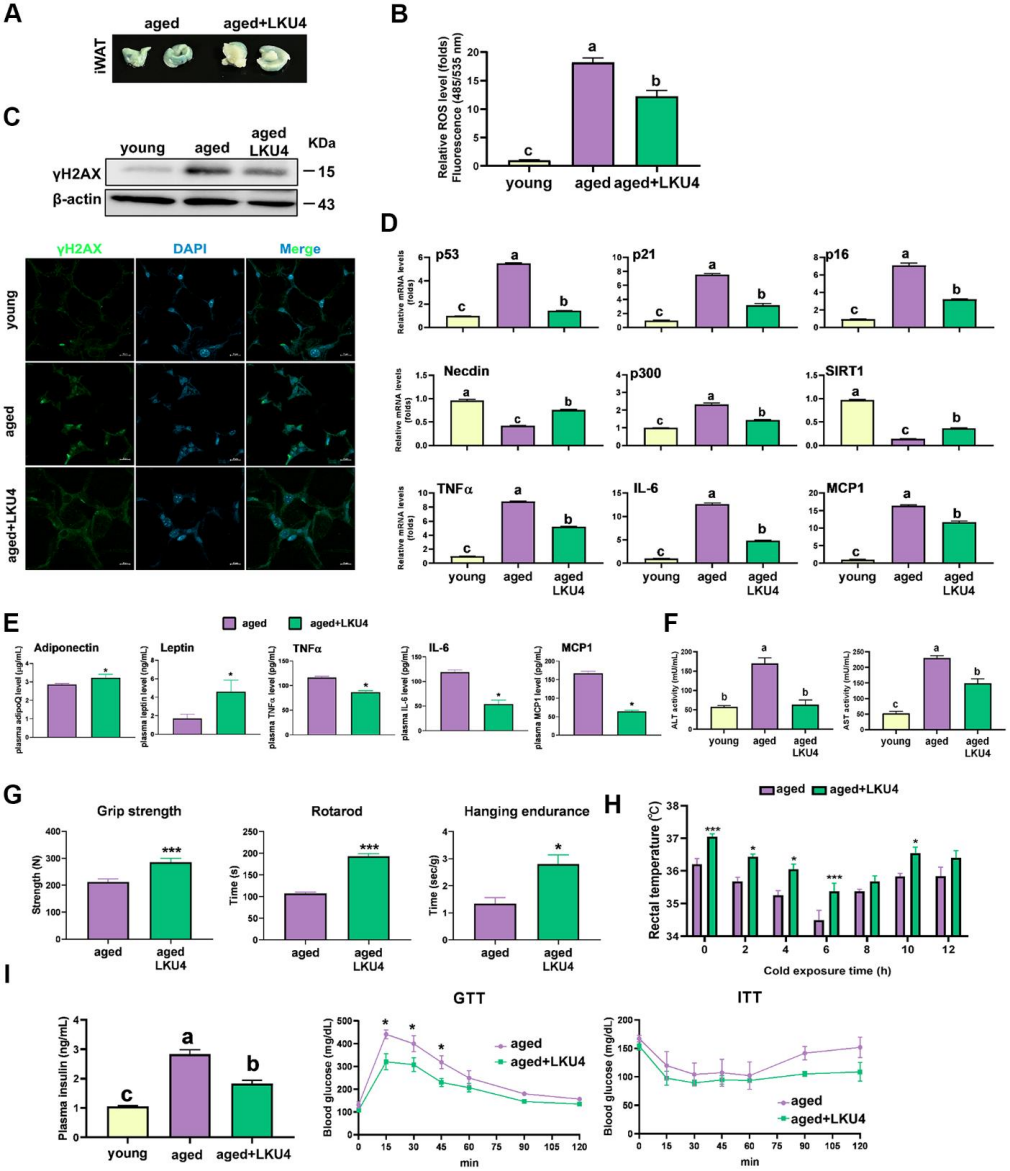
**Administration of LKU4 inhibits AT senescence during aging.** LKU4 or PBS was administered daily to 10-month-old C57BL/6J male mice for 10 or 14 months during feeding with a normal diet (ND). The 2-month-old ND-fed C57BL/6J male mice were designated as the young group. (**A**) SA-β-gal staining of inguinal WAT (iWAT) from aged (24-month) mice with or without LKU4 administration (n = 5). (**B**) Reactive oxygen species (ROS) levels in iWAT from each group of mice (n = 3). (**C**) Immunofluorescence staining images (green, γH2AX; blue, DAPI) and Western blot (WB) analysis of γH2AX in iWAT from each group (n = 3). Scale bars, 10 μm. (**D**, **E**) RT-qPCR analysis of cellular senescence-associated gene, SASP, and adipokine gene expression in iWAT (**D**) and plasma SASP and adipokine levels (**E**) of 2-month-old and 24-month-old mice (n = 3). (**F**) Plasma levels of alanine aminotransferase (ALT) and aspartate aminotransferase (AST) in each group of mice (n = 3). (**G**) Forelimb grip strength, rotarod, and hanging endurance tests in 20-month-old control and LKU4 mice (n = 5). (**H**) Rectal temperature was measured at 25° C (0 h) and 4° C for different durations (2–12 h) in 24-month-old mice (n = 8). (**I**) Plasma insulin levels, glucose tolerance test, and insulin tolerance test in 24-month-old mice (n = 7–8). All data are expressed as the mean ± S.E.M. * p < 0.05, *** p < 0.01. The lowercase letters above the graphs indicate statistical significance at p < 0.05.

A lower degree of staining for SA-β-gal was observed in the iWAT of 24-month-old LKU4 mice (aged LKU4 iWAT) compared to age-matched control iWAT (aged iWAT) (Figure 1A). Oxidative stress is widely implicated in aging, i.e., oxidative stress promotes DNA damage and cellular senescence. Therefore, the ROS levels were predictably much higher in the aged iWAT than in young iWAT (2-month-old mice); meanwhile, LKU4 administration decreased ROS generation in the aged iWAT by 34% (Figure 1B). Consistently, both the immunohistochemistry (IHC) and Western blot (WB) analyses showed that LKU4 markedly reduced γH2AX signals, a key indicator of DNA damage, in the aged iWAT (Figure 1C). Concomitantly, LKU4 administration also decreased mRNA levels of senescence marker genes, including *p53*, in the aged iWAT. Interestingly, the expression of *p300*, a key p53 coactivator, was increased in aged iWAT by about 2.2-fold, compared with that in the young iWAT. In contrast, the expression of p53 negative regulators (*SIRT1* and *NDN*) was reduced by ~56–86% (Figure 1D). However, these age-related gene expression changes were partially inhibited following LKU4 administration, suggesting that LKU4 mitigates age-mediated WAT senescence. Notably, WAT functions as an endocrine organ, secreting various adipokines, including cytokines; thus, age-related dysregulation of the adipose endocrine function contributes to the increased production of various SASP components, exacerbating local and systemic inflammation. Therefore, to determine whether LKU4 administration ameliorates SASP production, we performed an RT-qPCR analysis on iWAT. As expected, aging elevated the mRNA levels of SASP-related genes (*TNFα*, *IL-6*, and *MCP1*) by approximately 8–10-fold compared to those in the young iWAT. Subsequently, this age-related SASP mRNA levels were reduced in aged LKU4 iWAT by ~29–63%. Consistently, plasma levels of the TNFα, IL-6, and MCP1 in old mice were decreased by 27%, 55%, and 62%, respectively, following LKU4 administration (Figure 1E). In addition, LKU4 administration increased plasma adiponectin and leptin levels, which predominantly decline with age, implying that LKU4 aids in preserving the endocrine function of AT.

Notably, increased SASP production leads to chronic inflammation, contributing to age-related pathologies by impairing organ functions and systemic insulin sensitivity. Consistently, aged mice exhibited increased plasma levels of aspartate aminotransferase (AST) and alanine aminotransferase (ALT), liver damage markers, compared to young mice; meanwhile, LKU4 administration dramatically reduced these elevations (Figure 1F).

Next, we examined the exercise capacity and muscle strength to determine whether LKU4 affects age-related declines in physical function. Compared to age-matched control mice, LKU4 administration to 10-month-old mice for 10 months enhanced exercise performance capacity by ~1.3–1.8-fold, as measured using grip strength, rotarod, and hanging endurance tests (Figure 1G). Since aging is also associated with impaired thermoregulation [18], we assessed whether LKU4 could mitigate age-related hypothermia in 24-month-old mice. At room temperature (25° C), aged LKU4 mice showed mildly higher body temperature than control aged mice. Furthermore, upon cold exposure to 4° C, aged LKU4 mice consistently maintained a higher body temperature relative to control old mice at all measured time points (Figure 1H). Moreover, plasma insulin levels were increased with age (Figure 1I). However, LKU4 administration partially reduced this age-related increase in insulin levels. Furthermore, after intraperitoneal glucose injection, both aged control and aged LKU4 mice exhibited increased plasma glucose levels at 15 min, whereas a lower increase was observed in aged LKU4 mice. Although plasma glucose levels gradually declined in both mouse groups, aged LKU4 mice maintained significantly lower glucose levels than the aged control mice until 90 min after glucose injection. The insulin-induced glucose reduction was comparable in the insulin tolerance test between these mouse groups until 60 min post-insulin injection. However, the glucose levels in the control aged mice dramatically increased after 60 min, while the reduced plasma glucose levels were maintained in the aged LKU4 mice. These results indicate that LKU4 administration ameliorates adipose senescence and related aging phenotypes, such as inflammation, declined thermoregulation, and insulin resistance.

### LKU4 ameliorates age-related senescence of adipocytes in iWAT

**Figure 2:**
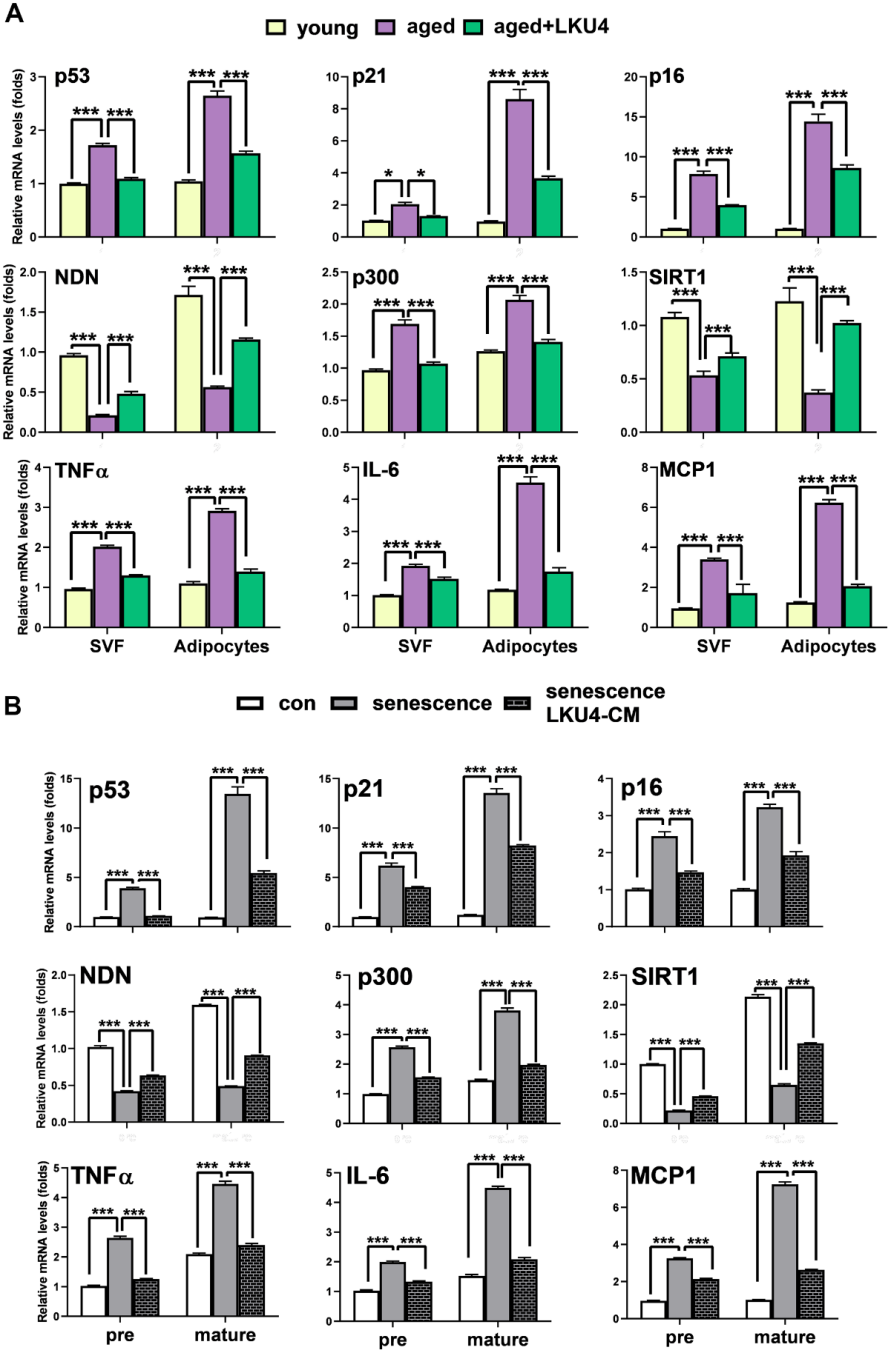
**LKU4 attenuates the susceptibility of adipocytes to age-related cellular senescence.** (**A**) RT-qPCR analysis of cellular senescence-associated gene expression in stromal vascular fraction (SVF) cells and adipocyte fractions from iWAT of 2-month-old and 24-month-old mice (n = 3). (**B**) RT-qPCR analysis of cellular senescence-associated gene expression in 3T3-L1 preadipocytes (pre) and day 8 3T3-L1 mature adipocytes (mature). Cells were treated with 100 μM H_2_O_2_ for 24 h in the absence or presence of LKU4–CM. All data are expressed as the mean ± S.E.M. * p < 0.05, *** p < 0.01.

Adipocytes constitute a major component of WAT and play a key role in WAT. Recent studies have shown that post-mitotic cells, such as mature adipocytes, can enter senescence [6]. Therefore, we next investigated the cell types that are more susceptible to age-related senescence in iWAT and more responsive to the LKU4-mediated inhibition of this process. Gene expression analysis of adipocytes and stromal vascular fraction (SVF) cells isolated from the iWAT of aged mice revealed that aging increased the expression of senescence-related genes (*p53*, *p21*, *p16*, and *p300*) while *NDN* and *SIRT1* expression decreased in both cell types (Figure 2A). Notably, adipocytes have higher mRNA levels of senescence-related genes than SVF cells. A similar expression pattern was observed for the SASP. However, upon LKU4 administration, these age-related changes in the gene expression profile were partially inhibited, with a more pronounced effect in adipocytes. Probiotic lactic acid bacteria (LAB) that reside in the gut promote health beneficial effects by secreting various signaling metabolites into circulation. However, only some of these signaling metabolites were reported from different strains, which include lactic acid, short-chain fatty acids, gamma-aminobutyric acid (GABA), and other signaling peptides [19–22]. Although the composition of metabolites produced by LAB, including LKU4, remains largely unknown, LAB-cultured CM have been used for *in vitro* studies to simulate the LAB-mediated effect on cell physiology. Thus, to further determine the effect of LKU4 on the senescence of preadipocytes and adipocytes, we treated 3T3-L1 cells with LKU4-CM. Consistently, when senescence was induced in 3T3-L1 preadipocytes and differentiated adipocytes following a 100 μM H_2_O_2_ treatment for 24 h, senescence-related genes were upregulated in both cell types, with a higher senescence effect observed in adipocytes (Figure 2B). In contrast, the expression of p53 negative regulators (*NDN* and *SIRT1*) decreased in these H_2_O_2_-treated cells. However, co-treatment of LKU4–CM concomitantly mitigated H_2_O_2_-induced upregulation of these senescence genes. Consistently, H_2_O_2_ treatment elevated the expression of SASP-related genes in both cell types, with a stronger effect in adipocytes. In line with the above findings, LKU4–CM partially reversed the H_2_O_2_-mediated induction of the SASP. These results indicate that adipocytes are more prone to age-related senescence in iWAT and that LKU4 has a suppressive effect on senescence for both adipocytes and SVF cells, with a higher inhibitory effect on adipocyte senescence.

### NDN regulates p53 acetylation and activity by releasing p300

**Figure 3:**
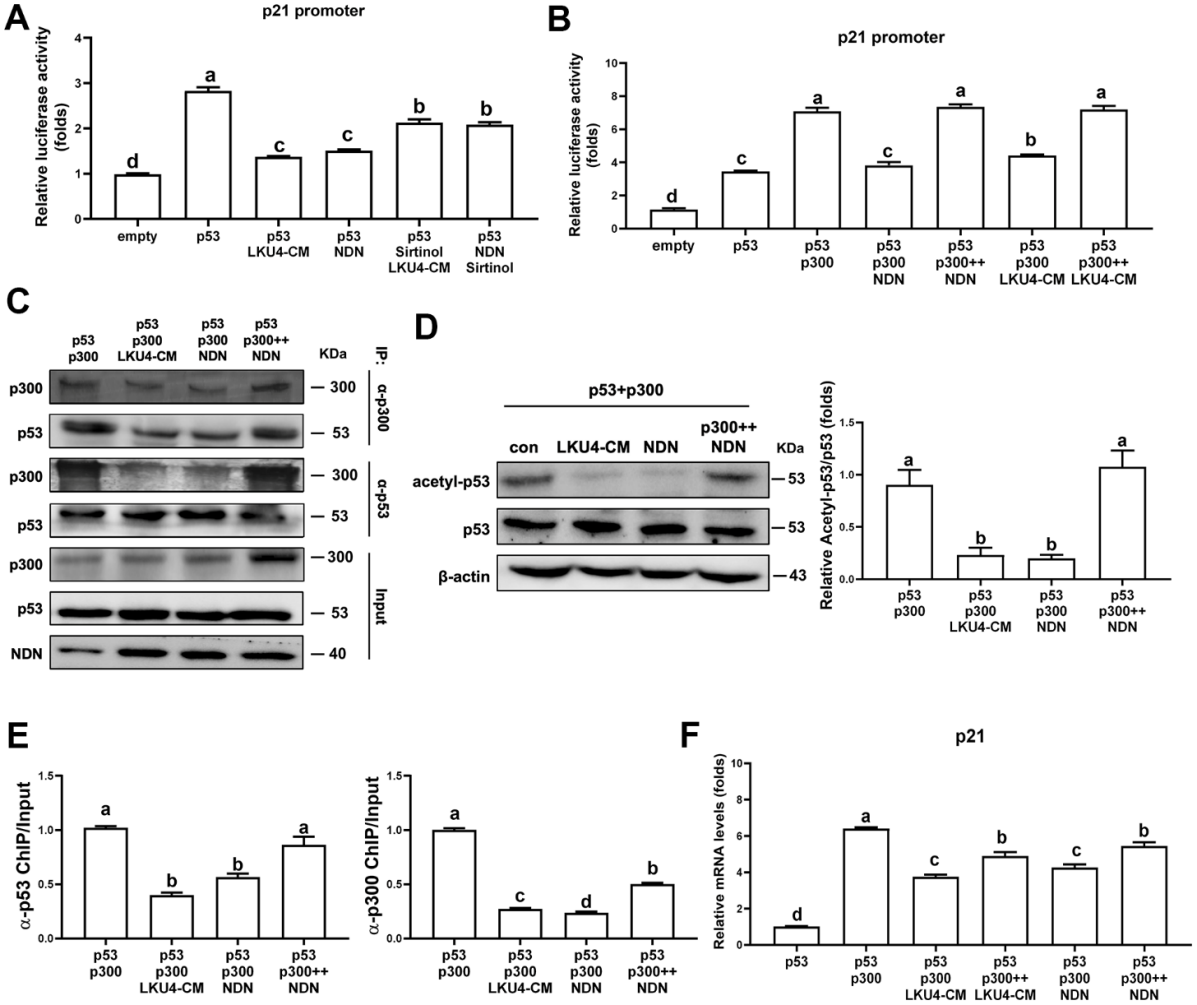
**Necdin regulates p53 acetylation and activity by releasing p300 from p53.** (**A**, **B**) Reporter gene analysis using p21-promoter–Luc and the indicated expression plasmids in HEK293T cells treated with 100 μM H_2_O_2_, 50 μM sirtinol, and LKU4–CM for 24 h. (**C**) Immunoprecipitation (IP) analyses of p53 and p300 in HEK293T cells transfected with p53, p300, and NDN expression plasmids with or without LKU4–CM treatment for 24 h. (**D**) p53 acetylation in day 8 3T3-L1 adipocytes transfected with the indicated plasmids and treated with LKU4–CM for 24 h. Cells were immunoprecipitated using an anti-p53 antibody. (**E**) Chromatin immunoprecipitation (ChIP) assay using anti-p53 and anti-p300 antibodies in day 6 3T3-L1 adipocytes transfected with p53, p300, and NDN, and treated with LKU4–CM for 24 h. (**F**) mRNA and protein levels of p21 in 3T3-L1 adipocytes transfected with p300 and NDN expression plasmids and treated with LKU4–CM for 24 h. The lowercase letters above the graphs indicate statistical significance at p < 0.05.

NDN is highly expressed in post-mitotic cells, such as neurons [23]. Consistently, NDN is predominantly expressed in the mature adipocytes of iWAT. A previous study demonstrated that NDN suppresses p53 transcriptional activity in post-mitotic neurons by promoting p53 deacetylation via SIRT1 recruitment [15]. Thus, since the gene expressions of *NDN* and *SIRT1* were reduced in adipocytes with age and were partially, but significantly, restored by LKU4 administration, we first determined whether LKU4 can suppress p53 transcriptional activity through the NDN–SIRT1 pathway using a reporter gene vector containing the p21 promoter (p21-promoter–Luc). As shown in Figure 3A, LKU4–CM and NDN reduced p53 activity in HEK293T cells by 40% and 39%, respectively. However, when sirtinol, a SIRT1-specific inhibitor, was administered alongside LKU4–CM or NDN, the observed suppressive effect on p53 activity was only partially released. This suggests that LKU4-induced NDN expression may modulate the p53 activity beyond its role as a SIRT1 recruiter. Previous studies have shown that NDN interacts with TAD2 (AAs: 33–62) in p53, which is also known to form part of the p300 binding sites [16]. Therefore, we examined whether NDN affects the p300-mediated coactivation of p53 transcriptional activity. As shown in Figure 3B, p300 significantly enhanced p53 induction of the p21 promoter activity in HEK293T cells, whereas LKU4–CM and NDN almost abolished the p300-mediated enhancement of the p53 activity. However, these inhibitory effects by LKU4 and NDN were reversed by increasing p300, suggesting that LKU4-induced NDN regulates p53 transcriptional activity by inhibiting the p300–p53 interaction. To investigate this possibility further, we performed *in vitro* immunoprecipitation (IP) assays in HEK293T cells overexpressing p53 and p300 using anti-p53 and anti-p300 antibodies. As expected, LKU4–CM or NDN treatment significantly reduced the interaction between p53 and p300 (Figure 3C). However, when the amount of p300 was increased in the HEK293T cells in the presence of NDN, the p53–p300 interaction was partially restored, indicating that NDN inhibits p53 activity by competing with p300 for binding to p53. Given that p300 enhances p53 activity by stimulating p53 acetylation, we examined the effect of LKU4–CM and NDN on p53 acetylation. Thus, p53 was first immunoprecipitated from 3T3-L1 adipocytes overexpressing p53 and p300 using an anti-p53 antibody, before determining the acetylation status using an anti-acetyl-lysine antibody. LKU4–CM and NDN reduced p53 acetylation by 52% and 51%, respectively, compared to the control adipocytes (Figure 3D). However, NDN-induced inhibition of p53 acetylation was reversed following the further addition of p300. Next, we performed chromatin immunoprecipitation (ChIP) assays in the 3T3-L1 adipocytes overexpressing p53 and p300 to determine whether NDN affects the recruitment of p300 and p53 to the mouse *p21* promoter region containing a p53 response element (P53RE). When NDN and LKU4–CM was added to 3T3-L1 adipocytes, p300 binding to the p21 promoter almost disappeared, accompanied by reductions of 61% and 45%, respectively, in p53 recruitment (Figure 3E). However, when additional p300 was introduced alongside NDN, p53 and p300 binding to the *p21* promoter was increased by 1.5- and 2.1-fold, respectively. Consistently, RT-qPCR analysis showed that p300 significantly increased *p21* mRNA levels in 3T3-L1 adipocytes; meanwhile, the co-addition of p300 with either LKU4–CM or NDN inhibited p300-induced *p21* mRNA levels by about 43% and 35%, respectively (Figure 3F). However, these inhibitory effects were partially reduced when an increased amount of p300 was introduced. These results suggest that LKU4 may inhibit *p21* expression in adipocytes by suppressing p300 coactivation of p53 transcriptional activity by inducing NDN expression.

### LKU4 negatively regulates H_2_O_2_-induced adipocyte senescence by upregulating NDN

**Figure 4:**
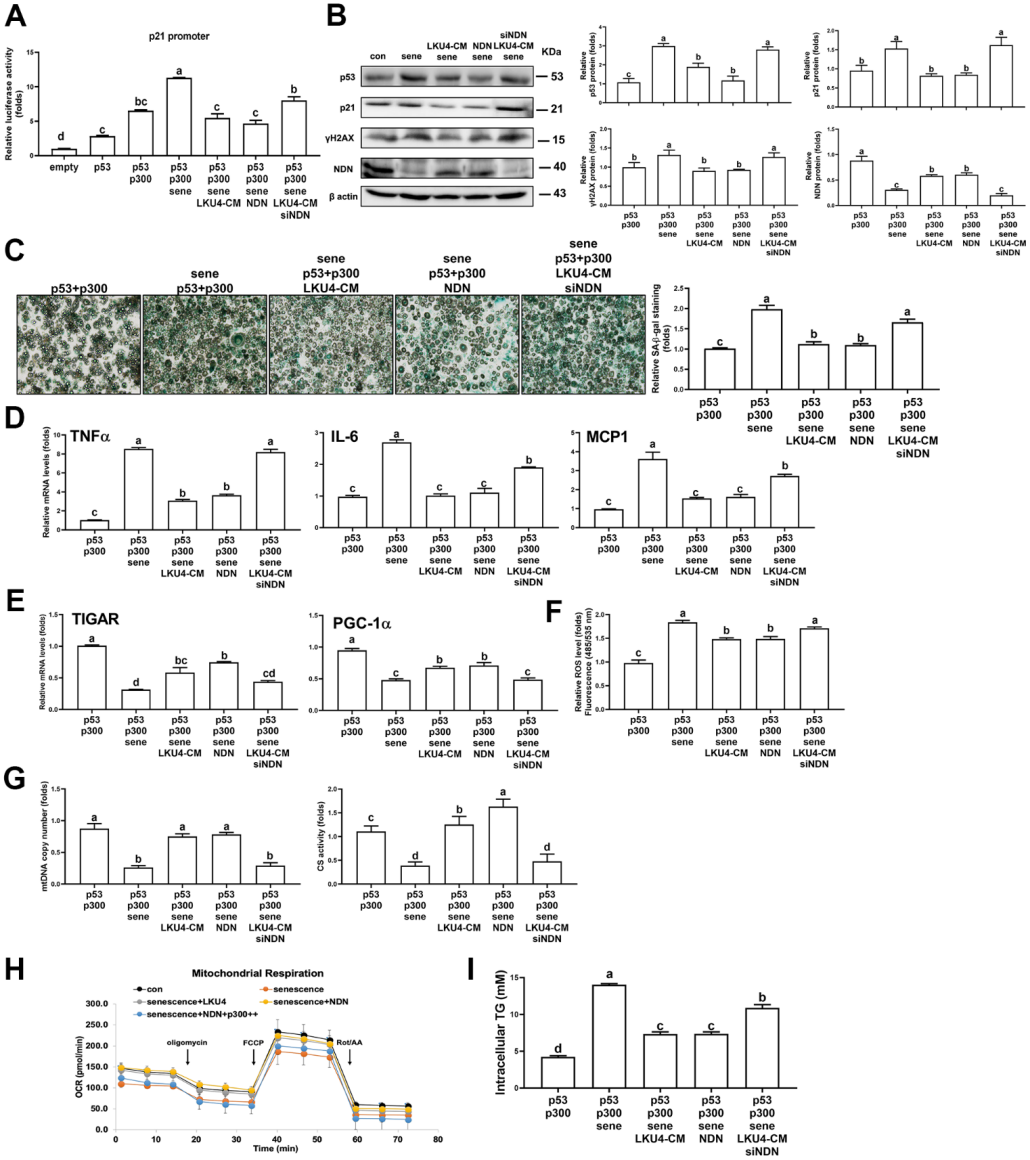
**LKU4 negatively regulates H_2_O_2_-induced adipocyte senescence through NDN upregulation.** (**A**) Reporter gene analysis using p21-promoter–Luc in HEK293T cells. (**B**–**G**) γH2AX, p53, p21, and NDN protein expression (**B**), SA-β-gal staining (scale bars, 200 μm) (**C**), RT-qPCR analysis of SASP genes (**D**) and mitochondrial function-associated genes (**E**), ROS levels (**F**), mtDNA copy number and CS activity (**G**) in primary adipocytes. Primary adipocytes differentiated from SVF cells were transfected with expression plasmids and NDN siRNA for 12 h, followed by treatment with 100 μM H_2_O_2_, 50 μM sirtinol, and LKU4–CM for another 24 h, as indicated, before analysis. (**H**) Oxygen consumption rate (OCR) analysis using a Seahorse XFe analyzer in 3T3-L1 adipocytes overexpressing the indicated expression plasmids in the absence or presence of LKU4–CM. (**I**) Intracellular TG levels in primary adipocytes. Differentiated primary adipocytes were transfected with expression plasmids and NDN siRNA and then treated with 100 μM H_2_O_2_, 50 μM sirtinol, and LKU4–CM, as indicated.

To determine the role of NDN in the LKU4-induced suppression of adipocyte senescence, we first performed a reporter gene assay in senescent HEK293T cells. Inducing senescence by administering 100 μM H_2_O_2_ for 24 h promoted p300 coactivation of *p53* transcriptional activity by 1.7-fold, compared to vehicle treatment (Figure 4A). However, when LKU4–CM or NDN was added to these cells, the effect of H_2_O_2_ on p300-induced p53 activity was reduced by 52% and 60%, respectively. In contrast, when *NDN*-specific siRNA (si*NDN*) was added alongside LKU4–CM, the inhibitory effect of LKU4 on H_2_O_2_-induced p53/p300 activity was almost abolished. Consistent with this observation, WB analysis showed that H_2_O_2_ treatment of adipocytes differentiated from SVF cells increased γH2AX, p53, and p21 levels by ~1.3–3-fold compared to vehicle treatment, in parallel with reduced NDN expression. However, LKU4–CM treatment reversed these H_2_O_2_-induced changes (Figure 4B). NDN also reduced γH2AX, p53, and p21 levels by ~50–60% in senescent adipocytes. Moreover, the co-addition of si*NDN* and LKU4–CM strongly inhibited the effect of LKU4 on these protein levels in senescent adipocytes. Subsequently, we performed SA-β-gal staining in H_2_O_2_-treated senescent adipocytes overexpressing p53 and p300. Both LKU4–CM and NDN decreased SA-β-gal staining in these senescent adipocytes, compared to the control senescent adipocytes. In contrast, NDN knockdown alongside LKU4–CM treatment abolished this observed LKU4 effect on SA-β-gal staining (Figure 4C). Consistently, both LKU4–CM and NDN inhibited H_2_O_2_-induced expression of the SASP (TNFα, IL-6, and MCP1) in adipocytes overexpressing p53 and p300; meanwhile, this inhibitory effect of LKU4–CM was attenuated by NDN silencing (Figure 4D). Notably, p53 is frequently associated with mitochondrial function in stress conditions, and an alteration in mitochondrial function plays a crucial role in cellular senescence, including oxidative stress and SASP production. Consistently, H_2_O_2_-induced senescence in adipocytes overexpressing p53 and p300 promoted a 69% reduction in the mRNA levels of genes involved in mitochondrial function (*TIGAR* and *PGC-1α*) (Figure 4E). These genes are also known as p53-regulated genes. Both LKU4–CM and NDN partially restored the expression of these genes in senescent adipocytes, while NDN knockdown abolished this LKU4–CM-induced restoration. Concurrently, when H_2_O_2_ was administered to adipocytes transfected with p53 and p300, the cellular ROS levels were 1.8-fold higher than those following vehicle treatment; meanwhile, LKU4–CM and NDN alleviated the H_2_O_2_ effect on the ROS levels in these cells by 12% and 14%, respectively (Figure 4F). However, when si*NDN* was introduced, the inhibitory effect of LKU4–CM on ROS generation was significantly reversed. In addition, H_2_O_2_-induced cellular senescence promoted a 70% and 65% reduction in mtDNA copy number and CS activity, respectively, in 3T3-L1 adipocytes overexpressing p53 and p300, compared to vehicle treatment (Figure 4G). However, these reductions were restored by both LKU4–CM and NDN treatments, whereas NDN knockdown in LKU4–CM-treated adipocytes abolished these observed LKU4-CM-mediated effects. Senescent cells are known to exhibit decreased ATP production with lower OCRs. In line with these observations, lower basal and maximal OCRs were observed in senescent 3T3-L1 adipocytes overexpressing p53 and p300. In contrast, both LKU4–CM and NDN treatments enhanced the basal and maximal OCRs in these senescent 3T3-L1 adipocytes (Figure 4H). Interestingly, increased p300 expression suppressed the basal and maximal OCRs, even in the presence of NDN. This decline in mitochondrial metabolism during aging is closely associated with decreased triglyceride (TG) utilization, thereby promoting excessive TG accumulation in the AT. Likewise, H_2_O_2_-treated senescent 3T3-L1 adipocytes overexpressing p53 and p300 exhibited 3.3-fold higher intracellular TG levels than untreated control 3T3-L1 adipocytes; meanwhile, both LKU4–CM and NDN reduced senescence-induced intracellular TG levels by 48% (Figure 4I). However, the senescence effect on intracellular TG levels was partially recovered when NDN was silenced alongside LKU4–CM treatment. These results indicate that the NDN-mediated regulation of the p53–p300 pathway is critical for attenuating senescence and restoring senescence-associated adipocyte dysfunctions.

### NDN knockdown abolished the inhibitory effect of LKU4 on age-related adipose senescence

**Figure 5:**
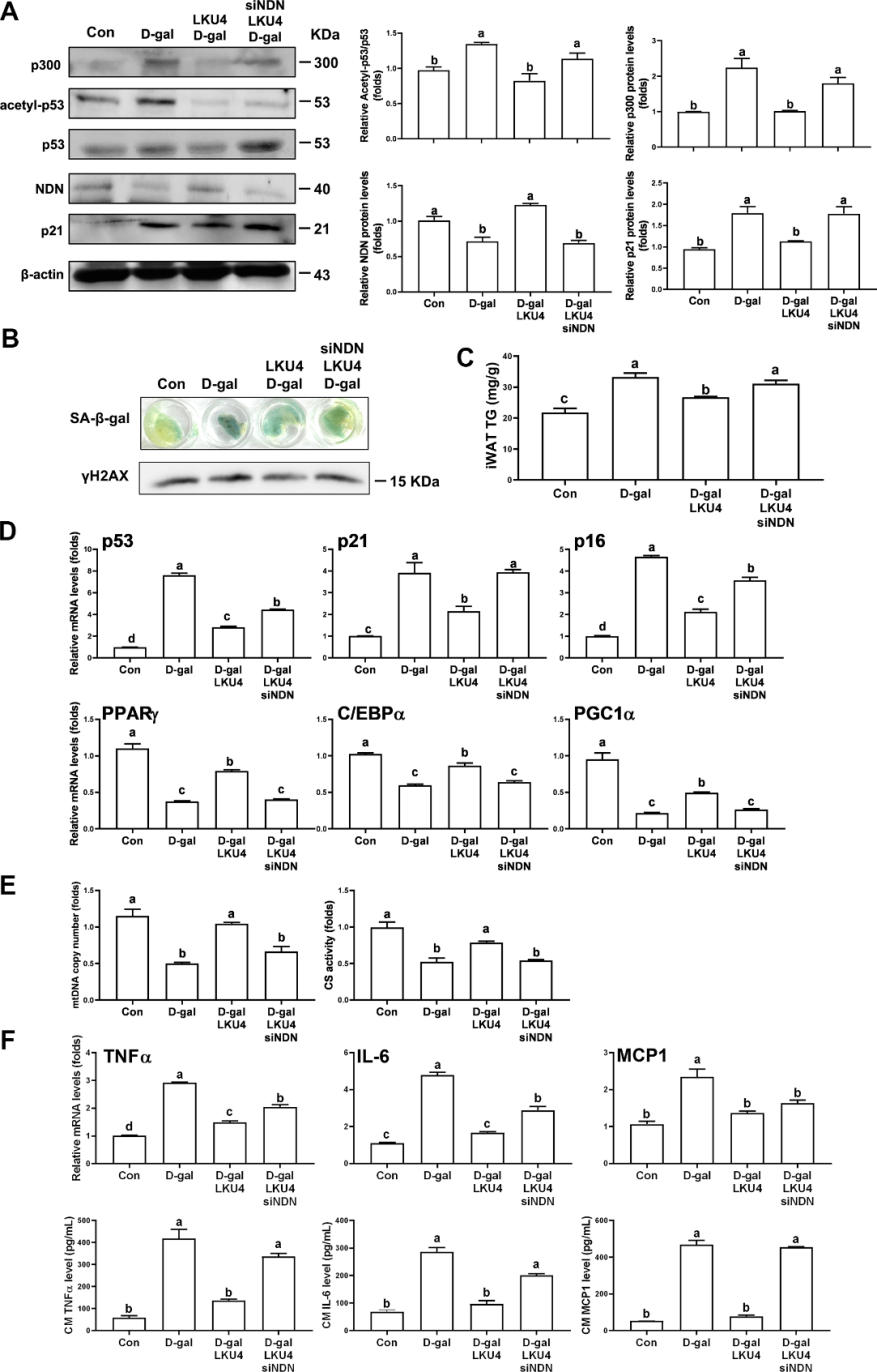
**NDN knockdown in *ex vivo* iWAT abolishes the inhibitory effect of LKU4 on age-related adipose senescence.** Eight-week-old C57BL/6J male mice were administered LKU4 or PBS daily for 4 weeks, followed by daily treatment of D-gal or 0.9% NaCl for an additional 8 weeks, with or without LKU4 supplementation. Mice treated with 0.9% NaCl, D-gal, and combined with LKU4 are designated as Con, D-gal, and D-gal/LKU4, respectively. Necdin was knocked down *ex vivo* in iWAT explants from D-gal/LKU4 mice using *necdin*-specific siRNA. (**A**) p53 acetylation in iWAT explants from each group of mice (n = 3). (**B**) SA-β-gal staining and protein levels of γH2AX in iWAT explants (n = 3). (**C**) Triglyceride (TG) levels in *ex vivo* iWAT explants from each group of mice (n = 3). (**D**) RT-qPCR analysis of senescence marker genes and lipid metabolism-related genes in iWAT explants (n = 3). (**E**) mtDNA copy number and CS activity (n = 3). (**F**) SASP mRNA levels in iWAT explants and SASP proteins secreted from iWAT explants (n = 3). The lowercase letters above the graphs indicate statistical significance at p < 0.05.

To further investigate the role of NDN in the protective effect of LKU4 on age-related adipose senescence, D-gal was administered daily to 12-week-old mice for 8 weeks to accelerate aging (D-gal mice). For the LKU4 administration group of the D-gal-aged mice (D-gal/LKU4 mice), we first administered LKU4 to mice for 4 weeks before D-gal treatment. The mice were then continuously administered LKU4 during the whole D-gal treatment period. Since LKU4 administration inhibited age-associated changes in senescence-related gene expressions in the iWAT, we performed WB analysis to determine the LKU4 effect on D-gal-induced adipose senescence in iWAT. As expected, D-gal treatment increased p300 and p21 expression in the iWAT by ~1.8–2.2-fold, and p53 acetylation by 1.4-fold compared to age-matched control mice, while NDN protein levels were reduced by 30%. LKU4 administration almost abolished D-gal-induced changes in these protein levels, as well as in p53 acetylation (Figure 5A). However, *ex vivo NDN* silencing in the iWAT explants from the D-gal/LKU4 mice (D-gal/LKU4 iWAT explant) using *NDN*-specific siRNA reversed the LKU4-mediated effect on p53, p300, and p21 protein levels and p53 acetylation, suggesting that NDN mediates the LKU4-induced inhibition of p53 activity.

Next, we examined whether *NDN* silencing attenuates the protective effect of LKU4 against adipose senescence in D-gal mice. Figure 5B shows increased SA-β-gal staining and γH2AX levels in *ex vivo* iWAT from D-gal mice (D-gal iWAT explant). These D-gal-induced changes were partially inhibited in D-gal/LKU4 iWAT explants, while *NDN* silencing restored the effects of D-gal in the SA-β-gal staining and the γH2AX levels in the D-gal/LKU4 iWAT explants. Furthermore, the TG levels were elevated by 1.5-fold in the D-gal iWAT compared to those in the iWAT of age-matched control mice (control iWAT). However, LKU4 reduced TG levels by 20% in the D-gal iWAT (Figure 5C). Conversely, *NDN* silencing in the D-gal/LKU4 iWAT explants increased adipose TG levels by 1.2-fold relative to the D-gal/LKU4 iWAT explants. These levels were comparable to those in the D-gal iWAT. Consistent with these findings, LKU4 administration reduced D-gal-induced mRNA levels of senescence marker genes (*p53*, *p21*, and *p16*) in the iWAT by ~45–63%, while increasing the mRNA levels of lipid metabolism-related genes (*PPARγ*, *C/EBPα*, and *PGC-1α*) in the D-gal iWAT by ~1.4–2.3-fold. Despite this LKU4-mediated protective effect against D-gal-induced changes in gene expression, *NDN* silencing restored the D-gal impact on the expression of these genes (Figure 5D). Considering that most of these genes are known to be regulated by p53, these data strongly suggest that NDN mediates the LKU4-induced protective effect on age-related adipose senescence by regulating p53 activity.

Mitochondrial function plays a crucial role in maintaining cellular homeostasis; thus, mitochondrial dysfunction is associated with impaired ATP production and cellular senescence. Therefore, to further elucidate the role of NDN as a key mediator of LKU4 inhibition in senescence-related mitochondrial dysfunction, we evaluated the mtDNA copy number and CS activity following *NDN* silencing in D-gal/LKU4 iWAT explants. Both mtDNA copy number and CS activity were reduced in D-gal iWAT explants by 47% and 48%, respectively, compared to iWAT explants from control mice (control iWAT explant) (Figure 5E). These reductions were partially restored in D-gal/LKU4 iWAT explants, whereas *NDN* silencing significantly reduced these LKU4 effects. Consistent with these results, the expression of SASP markers (TNFα, IL-6, and MCP1) was increased by ~2.3–4.8 fold in iWAT following D-gal treatment; meanwhile, LKU4 reduced the expression of these SASP markers by ~42–66% in *ex vivo* D-gal iWAT explants (Figure 5F). Moreover, *NDN* silencing restored the effect of D-gal on the expression of the SASP marker genes, which LKU4 suppressed. Consistently, when *ex vivo* D-gal iWAT explants were incubated in DMEM culture medium for 72 h, TNFα, IL-6, and MCP1 levels in the medium were ~4.2–8.8-fold higher than those from the control mice. However, these SASP levels were reduced by ~66–84% following incubation with the D-gal/LKU4 iWAT explants. However, *NDN* silencing in the D-gal/LKU4 iWAT abolished the inhibitory effect of LKU4 on D-gal-induced SASP production. These results demonstrate that NDN mediates LKU4-induced suppression of adipose senescence in D-gal mice by inhibiting p53 activity.

### NDN knockdown facilitates age-related functional changes of iWAT

**Figure 6:**
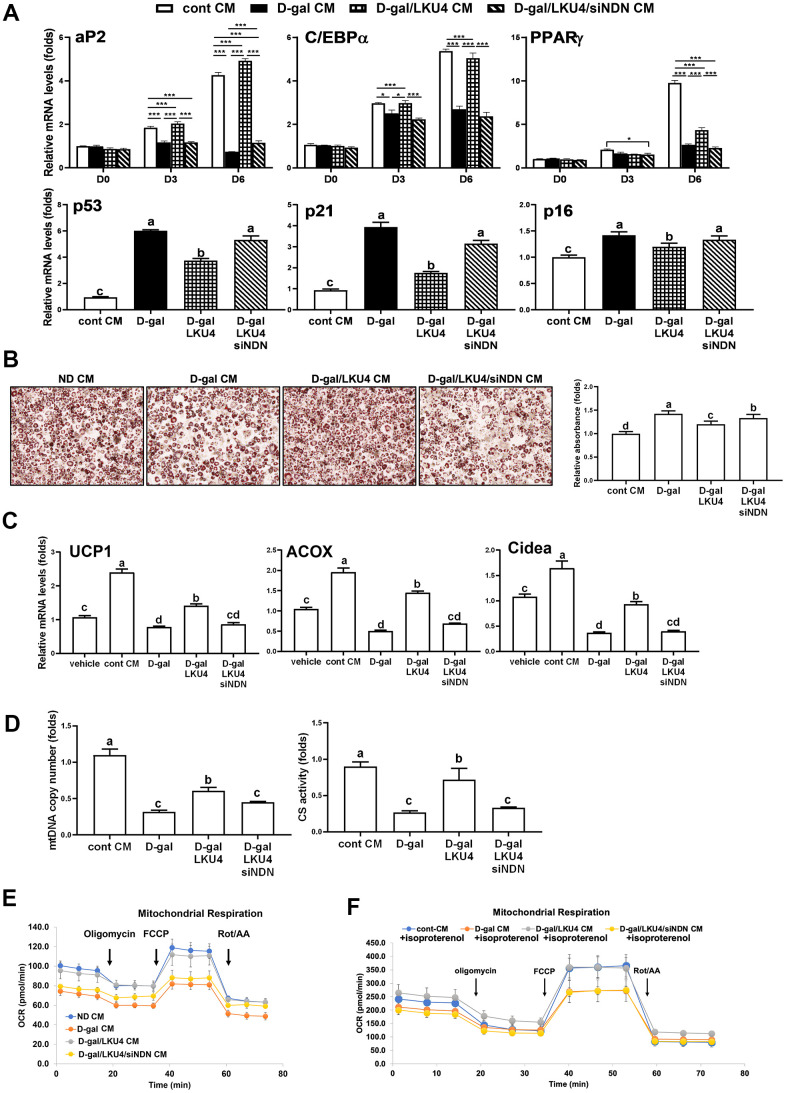
**NDN knockdown facilitates age-related changes in cell functions in iWAT.** (**A**–**E**) SVF and 3T3-L1 cells were differentiated in the absence or presence of CMs from iWAT explants. NDN knockdown was performed *ex vivo* in the iWAT explants from D-gal/LKU4 mice using *NDN*-specific siRNA. (**A**) RT-qPCR analysis of adipogenic genes during adipocyte differentiation. (**B**) Lipid accumulation was determined at day 6 by ORO staining. Scale bars, 200 μm. (**C**) On day 6, primary adipocytes differentiated from SVF cells were treated with 100 μM isoproterenol for 48 h. RT-qPCR was performed to analyze the expression of browning marker genes. (**D**) Mitochondria function was determined by measuring mtDNA copy number and CS activity in day 6 primary adipocytes. (**E**) OCR analysis of day 6 primary adipocytes and (**F**) OCR analysis of day 8 primary adipocytes treated with 100 μM isoproterenol for 48 h, were performed in the presence of oligomycin, FCCP, and rotenone/antimycin A using a Seahorse XFe analyzer at the indicated timepoints. All data are expressed as the mean ± S.E.M. * p < 0.05, *** p < 0.01. The lowercase letters above the graphs indicate statistical significance at p < 0.05.

AT undergoes consistent cellular remodeling by replacing old adipocytes with newly differentiated adipocytes to maintain its function. However, senescent adipocytes partially deteriorate this process by secreting SASP factors, which propagate senescence of neighboring adipose stem and progenitor cells. We observed that LKU4 administration reduced age-related SASP levels in plasma and AT in old mice. Thus, to determine whether LKU4 influences age-related senescence propagation in AT, we performed adipocyte differentiation of SVF cells in the presence of CM, which was incubated with each group of iWATs. To achieve this, we first prepared CMs from the iWATs of each group by collecting CMs after 72 h of incubation in DMEM culture media for control iWAT (cont CM), D-gal iWAT (D-gal CM), or D-gal/LKU4 iWAT (D-gal/LKU4 CM). Next, we isolated SVF cells from the AT of 3-month-old C57BL/6J mice. Then, we performed adipocyte differentiation of these cells for 6 days in the presence of CMs derived from the different iWAT explants, which provided a senescent microenvironment. When the SVF cells were differentiated in the presence of CM from the age-matched control AT, the mRNA levels of adipogenic marker genes (*aP2*, *C/BEPα*, and *PPARγ*) had gradually and substantially increased (Figure 6A). In contrast, the D-gal CM inhibited the expression of these adipogenic markers, while the D-gal/LKU4 CM inhibited the D-gal CM-induced suppression of adipogenic gene expression. However, this inhibitory effect by LKU4 was abolished following *NDN* silencing in the D-gal/LKU4 iWAT. Likewise, D-gal CM treatment increased the expression of senescence marker genes (*p53*, *p21*, and *p16*) in day 6 adipocytes by ~1.4–6.3-fold, compared to the control CM treatment. In contrast, the D-gal/LKU4 CM suppressed the D-gal CM-induced senescence gene expression. However, when the CM from the D-gal/LKU4 iWAT following *NDN* knockdown (D-gal/LKU4/siNDN CM) were used, the LKU4-induced inhibition of senescence marker genes was predominantly restored. In addition, ORO staining showed that adipocytes differentiated under D-gal CM exhibited reduced staining compared to those under control CM. However, when D-gal/LKU4 CM were used instead of the D-gal CM, the ORO staining of the adipocytes was similar to that of the control CM. Meanwhile, when *NDN* was silenced in the D-gal/LKU4 iWAT, this D-gal/LKU4 CM-mediated effect observed in the ORO staining disappeared, further confirming that aged AT can impair the adipogenicity of adipose progenitor cells and that NDN is essential for the inhibitory effect of LKU4 on adipogenic failure in aged WAT (Figure 6B). It is well known that the browning capacity of adipocytes in iWAT gradually declines with aging. Thus, to determine whether LKU4 influences age-related inhibition of adipocyte browning, we examined the effect of D-gal CM on the browning of adipocytes. Primary adipocytes differentiated from SVF cells were incubated for 48 h with each CM from different iWAT groups, followed by 100 μM isoproterenol treatment in the presence of each iWAT CM for another 48 h to induce adipocyte browning. As expected, adipocytes incubated in the control CM exhibited increased mRNA levels of browning marker genes (*UCP1*, *Cidea*, and *ACOX*) by ~1.6–2.4-fold, compared to vehicle-treated adipocytes, whereas the D-gal CM strongly inhibited the expression of these genes (Figure 6C). However, when the D-gal/LKU4 CM were used, the expression of these genes in adipocytes was increased by ~2.5–2.8-fold compared to the D-gal CM treatment. Nonetheless, when *NDN* was silenced in the D-gal/LKU4 iWAT, the LKU4 inhibition of the D-gal CM-mediated suppression of the browning gene expression was abolished. Consistently, the mtDNA copy number and CS activity in adipocytes treated with the D-gal CM were reduced by 63% and 70%, respectively, compared to those treated with the control CM (Figure 6D). However, the reduced mtDNA copy number and CS activity were partially restored when the D-gal/LKU4 CM were added instead. In contrast, this LKU4 effect on the D-gal CM-associated changes to the mitochondrial phenotypes was abolished when *NDN* was silenced in the D-gal/LKU4 iWAT. We also examined OCRs in primary adipocytes treated with CM from different groups of iWAT. The D-gal/LKU4 CM increased both the basal and maximal OCRs in adipocytes compared to the D-gal CM. However, when the CM from the D-gal/LKU4 iWAT with *NDN* silenced was used, the promoting effect of LKU4 on the basal and maximal OCRs under a senescence microenvironment was almost removed (Figure 6E). In addition, when isoproterenol was co-administered alongside the CM incubated with the iWAT from each group to determine the OCR under the adipocyte browning condition, the adipocyte OCRs in each condition showed a similar pattern to those observed in Figure 6E; however, their OCRs under isoproterenol treatment were much higher in each phase (Figure 6F). These results demonstrate that NDN is crucial for maintaining the physiology of adipose cells in a senescence microenvironment during aging.

## DISCUSSION

The beneficial functions of probiotics, including healthy aging, imply that probiotics perform regulatory roles in cellular senescence during aging. This study shows that LKU4 inhibits p53 acetylation in a SIRT1-independent manner by enhancing NDN expression, thereby preventing adipocyte senescence in AT of aged mice. This prevention improved adipocyte functions and suppressed the senescence of neighboring SVF cells. Moreover, senescence of post-mitotic adipocytes has been shown to enhance SASP secretion, exacerbating AT inflammation and promoting paracrine senescence transmission, impairing the differentiation ability of adipocyte progenitor cells [6].

AT is an energy storage and endocrine organ that secretes various adipokines, including adiponectin, leptin, and inflammatory cytokines. Among adipose tissue resident cells, adipocytes occupy a significant portion of AT and play a major role in the secretory function. Thus, age-related impairment of the adipocyte function, including low lipid storage capacity and increased SASP production, deteriorates the functions of other metabolic tissues, such as the liver and skeletal muscle, by promoting ectopic lipid accumulation, ultimately leading to systemic inflammation and metabolic disorders.

According to existing studies, various *Lactobacillus sp.* have been reported to promote healthy aging and longevity by suppressing oxidative stress, inflammation, and metabolic dysfunctions [24–27]. Although probiotics have anti-aging effects, the understanding of the mechanisms through which probiotics regulate the molecular pathways involved in cellular senescence during aging remains limited.

Although NDN has been known to promote neuronal differentiation and inhibit neuron apoptosis, the role of NDN in adipose physiology remains poorly understood. Accumulating evidence has revealed that NDN interacts with both p53 and SIRT1, and inhibits p53 acetylation by facilitating an association between p53 and SIRT1, suggesting that NDN negatively regulates p53 activity in a SIRT1-dependent manner to impede apoptosis in neurons [15].

Our study showed that NDN can inhibit p53 acetylation and p53 transcriptional activity in a SIRT1-independent manner since NDN suppression of p53 activity occurred even in the presence of sirtinol, a SIRT1-specific inhibitor. Furthermore, NDN directly suppresses p300 binding to p53, resulting in reduced p53 acetylation, which inhibits transactivation and binding to the *p21* promoter. Interestingly, p53 has two p300 binding sites: one (amino acids: 35–59) is in the p53 TAD2, which directly overlaps with a NDN binding region [16]. Therefore, NDN can suppress p53 function by promoting SIRT1 association with p53 and directly blocking p300 binding to p53. This indicates that the reduction in NDN in post-mitotic adipocytes during aging progressively induces p53 activity, promoting adipocyte senescence, which deteriorates adipose remodeling and disturbs the metabolic homeostasis by increasing the SASP. Consistently, we observed that NDN expression is reduced in SVF cells and adipocytes from aged iWAT, and LKU4 administration restored the age-associated decrease in NDN expression, more distinctly in adipocytes than in SVF cells. In parallel, LKU4 administration reduced age-related induction of senescence markers (p53, p21, and p16) in adipose cells, and this effect was more distinct in adipocytes. Although p53 levels in LKU4-treated senescent adipocytes were much lower than those in senescent adipocytes, the levels of p53 are comparable to those in normal adipocytes, suggesting that LKU4 may reduce cellular senescence and related aging phenotypes without enhancing the AT-related carcinogenic risk. Moreover, given the fact that NDN has been shown to be a putative tumor suppressor in many studies [28–31], the observation of increased NDN levels further strengthens that LKU4 safely reduces adipocyte senescence during aging. Furthermore, NDN silencing in the iWAT of LKU4-administered D-gal mice attenuated the LKU4 effect on p53 acetylation, senescence marker gene expression, and SASP production. These results indicate that NDN mediates the inhibitory effect of LKU4 on adipocyte senescence during aging. Additionally, NDN knockdown in the iWAT of D-gal LKU4 mice decreased the differentiation ability of SVF cells into adipocytes and induced mitochondrial dysfunction in primary adipocytes. Moreover, LKU4 administration improved insulin sensitivity and exercise performance in 24-month-old mice. Thus, adipocyte senescence during aging contributes to systemic insulin resistance by promoting lipid accumulation in the liver and skeletal muscle, suggesting that inhibition of adipocyte senescence by LKU4 potentially alleviates dysfunction in other metabolic tissues. Together, our findings demonstrate that LKU4 alleviates adipocyte senescence in the WAT of aged mice via NDN-mediated inhibition of the p53–p300 interaction in a SIRT1-independent manner, leading to improved age-associated metabolic abnormalities.

## MATERIALS AND METHODS

### Animals

Seven-week-old C57BL/6J male mice (weight 19 ± 2 g; Central Animal Laboratory, Daejeon, Korea) were acclimated for 1 week and then fed a normal diet (ND; 16% of the total calories obtained from fat; LabDiet, St. Louis, MO, USA) for 8 months under a 12 h light/dark cycle. LKU4 cells were cultured in MRS broth (BD Biosciences, San Jose, CA, USA) at 37° C for 24 h, before the cells were harvested at 4000 × g for 5 min; the pellets were resuspended in phosphate-buffered saline (PBS) at 1 × 10^8^ CFU/mL. A total of 200 μL of LKU4 resuspension or PBS was administered to the 10-month-old mice daily for 10–14 months. These mice were designated as the aged and aged + LKU4 groups, respectively. The young mice group included the 2-month-old ND-fed C57BL/6J male mice. Seven-week-old C57BL/6J mice were acclimatized for 1 week, and then fed a ND alongside the simultaneous oral administration of 200 μL of LKU4 resuspension or PBS for 4 weeks. D-galactose was dissolved in 0.9% NaCl solution, and 50 mg/kg D-galactose solution was administered via intraperitoneal injection for another 8 weeks. The mice were used for *ex vivo* AT transfection. The Institutional Animal Care and Use Committees at Chonnam National University (CNU-IACUC-YB-R-2022-26) approved all animal procedures.

### Plasmids

Plasmids, pBluescript II KS (+)–p21 promoter Luc (p21-promoter–Luc), were purchased from Addgene (Watertown, MA, USA). pCDNA3–p53 and pCDNA3–p300 were transfected into HEK293T cells, 3T3-L1 adipocytes or primary adipocytes. pCDNA3–NDN was constructed for a previous study.

### Cell culture, adipocyte differentiation, and senescence induction

The SVF cells were isolated from the inguinal WAT (iWAT) of 3-month-old male C57BL/6J mice, as described previously [14]. SVF cells were cultured and differentiated in Dulbecco's modified Eagle medium (DMEM) containing 10% fetal bovine serum (FBS). HEK293T cells and 3T3-L1 cells were cultured in DMEM containing 5% FBS or 10% newborn calf serum. On day 6 of differentiation, 3T3-L1 adipocytes and primary adipocytes differentiated from SVF cells were treated with 100 μM H_2_O_2_ or PBS for 24 h to induce cellular senescence. Cell-free supernatant was prepared by centrifugation after 24 h culture of LKU4 in MRS broth (BD Difco, Detroit, MI, USA) and used as LKU4 conditioned media (LKU4–CM). The LKU4–CM was added to the cells at a volume of 1/100 of the cell culture medium for 48 h. Sirtinol (50 μM, Sigma-Aldrich, St. Louis, MO, USA) and isoproterenol (100 μM, Sigma-Aldrich) were used in the experiments.

### RT-qPCR, chromatin immunoprecipitation (ChIP) analysis, Western blot (WB), and immunoprecipitation (IP) analysis

Total RNA was isolated from WATs, 3T3-L1 adipocytes, and primary adipocytes using RiboEx (GeneAll Biotechnology, Seoul, Korea), and cDNA synthesis was performed as previously described [32]. The real-time quantitative PCR (RT-qPCR) results of each target mRNA were calculated as relative values using the difference in Ct values between the target and *36B4* mRNA using the 2^-∆∆Ct^ method. The polymerase chain reaction (PCR) primer sequences are listed in Table 1. The ChIP assays were performed in 3T3-L1 adipocytes using α-p53 (Cell Signaling Technology, Danvers, MA, USA) and α-p300 antibodies (Santa Cruz Biotechnology, Santa Cruz, CA, USA). WB assays were performed using α-p53, α-p300, α-acetyl-lysine (Cell Signaling Technology), α-necdin (Abcam, Cambridge, UK), α-γH2AX (S139) (Abcam), p21 (Santa Cruz Biotechnology), and α-β-actin (Santa Cruz Biotechnology) antibodies, as described previously [33]. IP assays were performed using α-p53 and α-p300 antibodies, and WB was employed to analyze the immunoprecipitants.

**Table 1:** Primers used for the RT-qPCR.

**Gene**	**Sense primer: 5’–3’**	**Antisense primer: 5’–3’**
*p21*	AGTGCAAGACAGCGACAA	CGAGAACGGTGGAACTTTGAC
*p16*	GAACTCTTTCGGTCGTACCC	TGGGCGTGCTTGAGCTGA
*p53*	CCACCACACTATGTCGAAAAGT	ATGGCCATCTACAAGCAGTC
*NDN*	CATGATCCTGAGCCTCATCT	CGCTGGTACTTCAGGTAATT
*p300*	CAGTAGTGGACCAAATCAGGG	GAGAGCCCTGCTGTAGT
*Sirt1*	AGTTCCAGCCGTCTCTGTGT	CTCCACGAACAGCTTCACAA
*Acox*	TCGAGGCTTGGAAACCACTG	TCGAGTGATGAGCTGAGCC
*Tnfa*	AGCACAGAAAGCATGATCCG	CCCGAAGTTCAGTAGACAGAAGAG
*Il6*	ACCGCTATGAAGTTCCTCTC	CCTCTGTGAAGTCTCCTCTC
*Mcp1*	AGCACCAGCCAACTCTCAC	TCTGGACCCATTCCTTCTTG
*Tigar*	CACCAAGTGCTTGCAAGA	CAACATGGGTAACGGGATC
*Pgc1a*	GAGACTTTGGAGGCCAGCA	CGCCATCCCTTAGTTCACTGG
*Ucp1*	GGAGGTGTGGCAGTGTTC	TCTGTGGTGGCTATAACTCTG
*Pparg*	GAAGACCACTCGCATTCCTT	GAAGGTTCTTCATGAGGCCTG
*Cidea*	ATCACAACTGGCCTGGTTACG	TACTACCCGGTGTCCATTTCT
*36B4*	AGATGCAGCAGATCCGCAT	ATATGAGGCAGCAGTTTCTCCAG
*D-loop*	AATCTACCATCCTCCGTG	GACTAATGATTCTTCACCGT
*Gapdh*	GTTGTCTCCTGCGACTTCA	GGTGGTCCAGGGTTTCTTA

### Immunohistochemistry (IHC) staining

iWAT was isolated from each group of mice, fixed in 4% paraformaldehyde, and prepared as a paraffin-embedded section. The sections were stained with the α-γH2AX antibody, followed by the appropriate species-specific secondary antibody and DAPI. The specimens were imaged using LSM 900 confocal microscopy (Carl Zeiss, Oberkochen, Germany).

### Senescence-associated β-galactosidase (SA-β-gal) staining

iWATs, 3T3-L1 adipocytes, and primary adipocytes were incubated with X-gal in the staining solution, containing citric acid phosphate buffer (pH 6.0), 5 mM potassium ferricyanide, 5 mM potassium ferrocyanide, 150 mM NaCl, and 2 mM MgCl2, at 37° C for 24 h.

### Cellular reactive oxygen species (ROS) analysis, mitochondrial DNA, citrate synthase activity, and oxygen consumption rate (OCR) analysis

ROS levels were measured in iWAT and primary adipocytes using the DCFDA/H2DCFDA-Cellular ROS Assay kit (Abcam) according to the manufacturer’s protocol. Mitochondrial DNA (mtDNA) was isolated from 3T3-L1 adipocytes, primary adipocytes, and iWATs using a Genomic DNA Isolation kit (Qiagen, CA, USA), and the mtDNA copy number was analyzed by RT-qPCR using mtDNA primers. Citrate synthase (CS) activity was measured in 3T3-L1 adipocytes, primary adipocytes, and iWAT using a Citrate Synthase Activity Assay kit (BioVision, Milpitas, CA, USA). Oxygen consumption rate (OCR) analysis was performed using the Seahorse XF Cell Mito Stress Test kit (Agilent, Santa Clara, CA, USA) and the Seahorse Xfe96 analyzer (Agilent), as previously described [14]. OCR data were normalized by conducting *in situ* cell counting using BioTek Cytation 5 (Agilent).

### SASP analysis

Adiponectin (Invitrogen, Waltham, MA, USA), leptin (Invitrogen), TNFα (Invitrogen), IL-6 (R&D Systems, Minneapolis, MN USA), and MCP1 (R&D Systems) levels were measured in either the mouse plasma or supernatant of 3T3-L1 and primary adipocytes using ELISA kits.

### Statistical analysis

All data are expressed as the mean ± S.E.M. Statistical analysis was performed by Tukey’s multiple comparison test using SAS software (Version 9.4, SAS Institute) or Student’s t-test. A p-value < 0.05 was considered statistically significant. All the experiments were performed at least in triplicate.
